# Steroidogenic effects of *Taraxacum officinale* extract on the levels of steroidogenic enzymes in mouse Leydig cells

**DOI:** 10.1080/19768354.2018.1494628

**Published:** 2018-11-08

**Authors:** Hyun Joo Chung, Yoohun Noh, Min Su Kim, Ara Jang, Chae Eun Lee, Soon Chul Myung

**Affiliations:** a Department of Urology, Chung-Ang University College of Medicine, Seoul, Republic of Korea; b Advanced Urogenital Diseases Research Center, Chung-Ang University College of Medicine, Seoul, Republic of Korea; c Bio-Integration Research Center for Nutra-Pharmaceutical Epigenetics, Chung-Ang University, Seoul, Republic of Korea; d Department of Anatomy and Cell Biology and Neurology, College of Medicine, Chung-Ang University, Seoul, Republic of Korea; e Department of Urology, Seoul Medical Center, Seoul, Republic of Korea

**Keywords:** *Taraxacum officinale* extract (TOE), medicinal plant, steroidogenesis, late-onset hypogonadism (LOH), Leydig cell

## Abstract

In this study, we investigated the steroidogenic effect of *Taraxacum officinale* extract on mouse TM3 Leydig cells, which produce male hormones by increasing the levels of steroidogenic enzymes. Steroidogenic enzymes are involved in the production of testosterone in the testis. To date, the steroidogenic effect of *T. officinale* has not been reported. Therefore, we examined the steroidogenic effects of *T. officinale* extract (TOE) on mouse Leydig cells in vitro. Traditionally, plants have been used for the treatment of various kinds of ailments. For many years, some medicinal plants have been used to regulate steroidogenesis or late-onset hypogonadism (LOH). In particular, plants belonging to the genus Taraxacum have anti-inflammatory, anti-nociceptive, anti-oxidant, and anti-cancer properties. In this study, we determined whether the TOE exerts steroidogenic effects by increasing the levels of enzymes associated with steroidogenesis, such as the steroidogenic acute regulatory protein (STAR), CYP11A1, and translocator protein (TSPO) in the mitochondria and CYP17A1 in the smooth endoplasmic reticulum, in mouse Leydig cells. Our results showed that the TOE significantly increased the mRNA and protein levels of steroidogenic enzymes, thereby increasing the testosterone levels in mouse Leydig cells. Thus, our results indicate that the TOE increases the levels of steroidogenic enzymes, and further studies are required to establish the potential of this plant in regulating steroidogenesis and improving LOH.

## Introduction

Testosterone plays an important role in spermatogenesis and maintenance of secondary sexual functions in men. Male hypogonadism, also known as testosterone deficiency syndrome (TDS), is often associated with impaired puberty, impotence, gynecomastia, and infertility or a decrease in spermatogenesis (Wu et al. 2000). A previous longitudinal study on age-related changes in serum testosterone levels showed that the incidence of hypogonadism in men increased with age (Feldman et al. 2002). Testosterone is principally produced in the testicular Leydig cells under the regulation of the luteinizing hormone (LH) and then is released into the blood (Miller 1988). Male hormones are produced by fetal Leydig cells (FLCs), and fetal Sertoli cells masculinize the male embryos (Wainwright and Wilhelm 2010; Svingen and Koopman 2013; Wen et al. 2016). Leydig cells were isolated and identified for the first time in 1850 by Franz Leydig; these cells are present in fetal and adult testes (Wen et al. 2016). The Leydig cells produce male hormones, and the production of androgen is stimulated by binding of LH to its receptor (LH-R), which activates adenylyl cyclase and increases the production of cAMP and cAMP-dependent protein kinase. This signal activates the steroidogenic acute regulatory (STAR) protein and translocator protein (TSPO) in the inner membrane of the mitochondria, a critical step for the initiation of steroidogenesis in the testis. The substrate cholesterol is metabolized by cytochrome P450 cholesterol side-chain cleavage enzyme (P450scc) to pregnenolone, which is followed by metabolization of the enzymes, 3β-hydroxysteroid dehydrogenase (3β-HSD), cytochrome P450 17α-hydroxylase/C17-20 lyase (P450c17), and 17β-hydroxysteroid dehydrogenase (17β-HSD), which results in the production of testosterone (Beattie et al. 2015).

The production of testosterone is decreased in aged Leydig cells, because of cellular changes in the steroidogenic pathway that decrease the production of testosterone, decrease LH stimulated cAMP production and downregulate STAR, CYP11A1 in the mitochondria, and CYP17A1 in the smooth endoplasmic reticulum (Zhong et al. 2013; Beattie et al. 2015; Ohta et al. 2017).

Therefore, preventing the age-dependent decrease in testosterone production in Leydig cells may provide a variety of benefits, which are clinically significant for improving libido and sexual function, fertility, bone density, muscle mass, and quality of life of aging men (Decaroli and Rochira 2016; Cormier et al. 2017). However, direct injection of artificial testosterone or oral testosterone administration can simultaneously induce many side effects, including prostate cancer, benign prostatic hyperplasia, and cardiovascular events (Grober 2014). Moreover, aging is a long process, and the long-term application of pharmacological levels of testosterone is limited by its potential side effects on health. Therefore, we have attempted to identify natural compounds present in food and food supplements that have fewer side effects and which effectively increase testosterone biosynthesis in Leydig cells.

A plant belonging to the genus Taraxacum, commonly known as ‘dandelion’ in English-speaking countries as ‘dent-de-lion’ from in French, has been used as folk medicine for a long time. The name Taraxacum has been derived from the Greek words ‘taraxis’ for inflammation and ‘akeomai’ for curative (Sweeney et al. 2005; Schutz et al. 2006). In particular, the species *Taraxacum platycarpum*, *Taraxacum ohwianum*, *Taraxacum hallaisanense*, *Taraxacum coreanum*, and *T. officinale* are found in South Korea (Lee et al. 2013). Taraxacum extracts and their constituents have anti-inflammatory, anti-nociceptive, anti-oxidant, anti-cancer activities in breast and uterine cancers (Jeon et al. 2008; Choi et al. 2010; You et al. 2010; Lee et al. 2012; Hfaiedh et al. 2016). In a previous study, we showed that extracts from dandelion plants effectively improved late-onset hypogonadism (LOH) clinically in men and enhanced physical performance and activation of spermatogenesis in rats. These effects may be attributed to protection and activation of Leydig cells and the production of endogenous testosterone (Noh et al. 2012). The mechanism underlying the steroidogenic effect of *T. officinale* extract (TOE) in mouse Leydig cells has not been studied thus far.

In this study, we examined whether the TOE could increase the biosynthesis of testosterone directly in Leydig cells in vitro. Furthermore, we investigated the mechanism underlying the TOE-mediated increase in the expression of STAR, CYP11A1, and CYP17A1 genes in mouse Leydig cells by monitoring the mRNA, protein, and testosterone levels.

## Materials and methods

### Plant extract

#### Plant material: dandelion

The fresh *T. officinale* was collected from the local market (Eumseong-gun, Chungcheongbuk-do, Korea). All samples were washed under tap water and dried in an oven at 40°C for 3 days. The plant materials were put through a grinder with a mesh size of 2 mm.

#### Preparation of extract

For extract preparation, dry plant materials were extracted twice with a 10-fold volume of water for 3 h by refluxing then filtered (Whatman, Little Chalfont, UK). After filtration, aqueous extracts were concentrated under reduced pressure, by using a rotary vacuum evaporator (Eyela CCA-1111, Tokyo Rikakikai Co., Ltd., Tokyo, Japan) and lyophilized (Bondiro; IlShin BioBase, Seoul, Korea) to obtain powder.

### Cell culture

TM3, mouse Leydig cells are derived from 11 to 13 days old mouse testes (ATCC No CRL – 1714, Manassas, USA). They were cultured in DMEM (Dulbecco’'s modified Eagle’'s medium) supplemented with 100 units/mL penicillin, 100 µg/mL streptomycin and 5% fetal-bovine serum at 37°C under a 5% CO_2_ atmosphere. Every 2 days, the media was changed before experiments.

### Cell viability assay

To assess the effects of TOE on TM3 Leydig cell viability, MTT assay kit (cell counting kit-8, CK04) was purchased from Dojindo Molecular Technologies, INC. (Rockville, USA) and was done according to the manufacturer's instrument. A density of 5.0 × 103 cells/well was seeded on each 96 well plate after then the assay was done at 12 and 48 h. The absorption was read at a wavelength of 450 nm and all experiments were performed in triplicate.

### Quantitative real-time reverse transcriptase-PCR (qRT-PCR)

TM3 cells were plated in six-well plates at a density of 5.0 × 105 cells/well and cultured with DMEM (5% FBS) for 24 h. Changing media into DMEM with 1% FBS and after incubation for another 24 h in the absence or presence of TOE (0, 1, 10, 25 and 50 µg/ml). Total RNA was extracted from the cells using the RNeasy mini kit (Qiagen, Germany) according to the manufacturer's instructions. Total RNA was reverse-transcribed using a high capacity cDNA reverse transcription kit, QuantiTect^®^ Reverse Transcription kit (Qiagen, Germany). Real-time quantitative PCR were done with the Rotor-Gene^®^ SYBR^®^ Green PCR kit (Qiagen, Germany).

Analysis was performed using the Rotor-Gene Q PCR machine with 2 step cycling protocol as denaturation was done at 95°C for 5 s and annealing/extension step was performed 60°C for 10 s and required 40th cycles (Qiagen, Germany). The cycle threshold (*C_t_*) values are similar to within 0.1 among triplicates. The primers were designed for CYP11A1, CYP17A1, STAR and 18s rRNA, which yielded a 2-ΔΔCt value and used for normalization. 18s rRNA was used as an internal marker. Primers were designed with the following oligonucleotides: CYP11A1-F (5′-ACA TGGCCAAGATGGTACAGTTG-3′), CYP11A1-R (5′ACGAAGCACCAGGTCATTCAC-3′), CYP17A1-F (5′-CTCCAGCCTGACAGACATTCTG-3′), CYP17A1R (5′-TCTCCCACCGTGACAAGGAT-3′), STAR-F (5′-TCT CTAGTGTCTCCCACTGCATAGC-3′), STAR-R (5′-TTAGCATCCCCTGTTCG AGCT-3′),18s rRNA-F (5′-GAG GCCCTGTAATTGGAATGAG-3′) and 18s rRNA-R (5′-GCAGCAACTTTAATATACGCTATTGG-3′). The experiments were performed in triplicate.

### Western blot analysis

For western blot analysis, cells were harvest in a 10 mM Tris (pH 7.4) buffer containing 1% SDS and 1 mM Na3VO4 on ice and 20 µg of cell lysate were separated by 10% SDS-PAGE and electro-transferred onto a nitrocellulose membrane as described elsewhere. The blots were then washed in Tris-buffered saline (20 mM Tris-HCl, pH 7.6 containing 137 mM NaCl and 0.05z (vWv) Tween 20), blocked with 5% skim milk for 1 h, incubated with primary antibody (1:1000) at 4°C for overnight. Primary antibodies used for immunoblotting were anti-actin (Santa Cruz, USA; #sc-47778), anti-CYP11A1 (Abcam, USA; #ab175408), anti-CYP17A1 (Abcam, USA; #ab125022) and anti-STAR (Abcam, USA;#ab203193). After then the membranes were rinsed with TBST three times (10 min each) and then incubated with secondary antibody (1:5000) labelled with horseradish peroxidase (HRP) at room temperature for 1 h. After the reaction the membranes were rinsed with TBST three times (10 min each), the signals were detected using the enhanced chemiluminescence Western blot kit (EzWestLumi plus) from ATTO Corporation (Motoasakusa, Tokyo, Japan) and analyzed using the ImageQuant LAS 4000 mini (GE Healthcare) machine. The experiments were performed in triplicate and data were quantified by Image J program.

### ELISA (enzyme-linked immunosorbent assay) for detecting testosterone

To detect the testosterone from cell supernatant, TM3 cells were plated in dish and cultured with DMEM (5% FBS) for 24 h. Changing media into DMEM with 1% FBS and after incubation for another 48 h in the absence or presence of TOE (0, 1, 10, 25 and 50 µg /ml). The cell supernatants were freezing before the experiment. ELISA procedure were done according to the manufacturer's instrument (R&D Systems^®^, Inc, Parameter TM Testosterone Assay, # KGE010). Detection was by measurement of the absorbance at 450 nm. The experiments were performed in triplicate.

### Statistical analysis

All data are presented as the mean ± standard deviation (S.D.). Differences between groups were determined with a one-way analysis of variance (ANOVA) followed by the LSD multiple comparison test, using SPSS version 20.0 for Windows and considered statistically signiﬁcant when *p* < .05 is marked with an asterisk (*) and *p* < .001 is marked with ** on each graph.

## Results

### HPLC chromatogram of TOE

**Figure 1.**
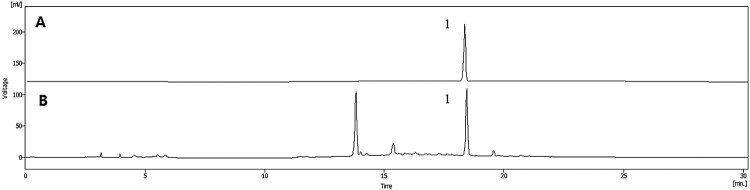
HPLC chromatograms of standard sample (A) and *Taraxacum officinale* extract (B). Peak1, Chicoric acid. The compound in *T. officinale* was identified on the basis of the retention time and chromatogram pattern, by co-chromatography with an authentic standard. From the data presented, peak 1 with a retention time of 18.45 min, was chicoric acid. The contents of chicoric acid in our study had high level of around 2.6 mg/g.

TOE was prepared using dried dandelion. We identified the compound as *T. officinale* on the basis of the retention time and chromatogram pattern (Figure 1(B)) and by cochromatography using an authentic standard (Figure 1(A)). Peak 1 with a retention time of 18.45 min was identified as chicoric acid (Figure 1). Our extract has high levels of chicoric acid 2.6 mg/g (Figure 1).

### TOE doesn’ t effect on cell viability with mouse TM3 Leydig cells

**Figure 2.**
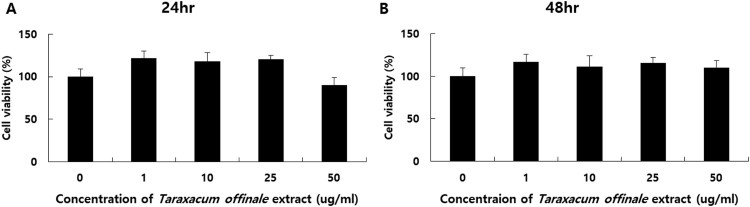
Cell viability of TM3 Leydig cells treated *Taraxacum officinale* extract. The varying concentration of 0, 1, 10, 25 and 50 µg/ml of *T. officinale* were treated into TM3 cells for 24(A) and 48 h(B). The experiments were performed in triplicate.

To investigate whether TOE effect on cell viability, we treated with varying concentration of *T. officinale* as 0, 1, 10, 25, and 50 µg/mL for 12 and 48 h on mouse TM3 cells. After MTT assay, TOE didn’t have an effect on cell viability significantly (Figure 2).

### TOE induces mRNA expression of steroidogenic genes in mouse TM3 Leydig cells

**Figure 3.**
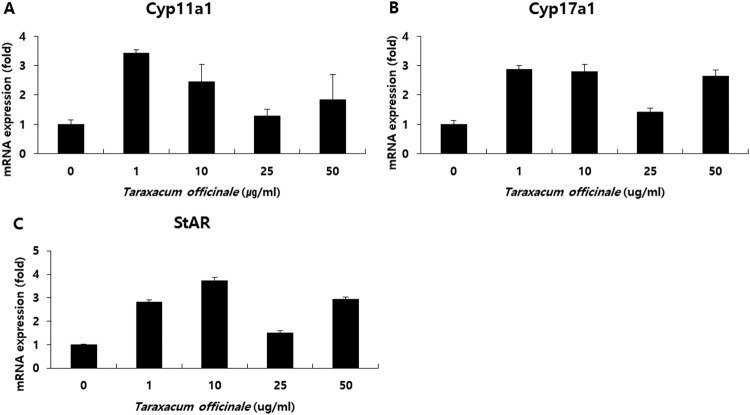
Measurement of mRNA levels of Cyp11a1, Cyp17a1 and StAR in mouse Leydig cells. Cells were treated with 0, 1, 10, 25 and 50 µg/ml of *T. officinale* for 24 h. The qRT-PCR was performed with the gene-specific primers for Cyp11a1 (A), Cyp17a1 (B) and StAR (C). mRNA were normalized with 18s rRNA gene. The experiments were performed in triplicate.

We treated mouse Leydig cells TM3 with 0, 1, 10, 25, and 50 µg/mL of TOE for 24 h with 1% fetal-bovine serum (FBS) in Dulbecco’s modified Eagle’s medium (DMEM). Then, we harvested the total RNAs and performed real-time PCR. The cycle threshold (*C_t_*) values were similar to within 0.1 among triplicates. The primers were designed for CYP11A1, CYP17A1, STAR, and 18s rRNA, which used for normalization. We examined the mRNA levels of the steroidogenic genes STAR, CYP11A1, and CYP17A1; our results showed that TOE increased the expression of these three genes. The expression of CYP11A1 increased approximately 3.5 times after treatment with 1 µg/mL of TOE (Figure 3(A)), that of CYP17A1 increased approximately 3 times after treatment with 1 µg/mL of TOE (Figure 3(B)), and that of STAR increased approximately 4 times after treatment with 10 µg/mL of TOE (Figure 3(C)) compared to the expression of these genes in the control group (0 µg/mL). Thus, treatment with TOE at specific dosages activated the steroidogenic enzymes CYP11A1, CYP17a1, and STAR and increased their mRNA levels in vitro. Therefore, TOE had an effect on steroidogenesis in the Leydig cells of male mice (Figure 3).

### TOE increases the expression of proteins associated with steroidogenic genes in mouse Leydig cells

**Figure 4.**
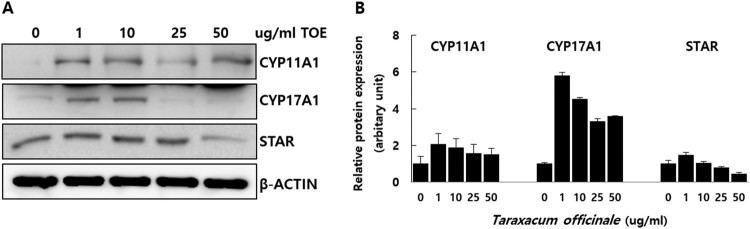
Measurement of protein levels of CYP11A1, CYP17A1 and STAR in mouse TM3 Leydig cells. Cells were treated with 0, 1, 10, 25 and 50 µg/ml of *T. officinale* for 48 h. The Western blotting analysis was performed with the gene-specific antibodies for CYP11A1, CYP17A1 and STAR (A). Total protein were normalized with ß-ACTIN. The data were quantified by using Image J program (B). The experiments were performed in triplicate.

We treated mouse TM3 Leydig cells with 0, 1, 10, 25, and 50 µg/mL of TOE for 48 h with 1% FBS in DMEM; the cells were harvested and the cellular protein was obtained. Subsequently, we performed western blotting analysis. We used 20 µg of the protein sample and the protein levels were detected using anti-STAR, anti-CYP11A1, and anti-CYP17A1 antibodies. The expression of STAR, CYP11A1, and CYP17A1 proteins were detected, and our results showed that treatment with TOE (10 µg/mL) increased the expression of these proteins (Figure 4). Thus, TOE (10 µg/mL) activated the steroidogenic enzymes CYP11A1, CYP17a1, and STAR and increased their protein levels in vitro. Therefore, TOE is associated with steroidogenesis with mouse Leydig cells (Figure 4).

### TOE increases testosterone synthesis in mouse Leydig cells

**Figure 5.**
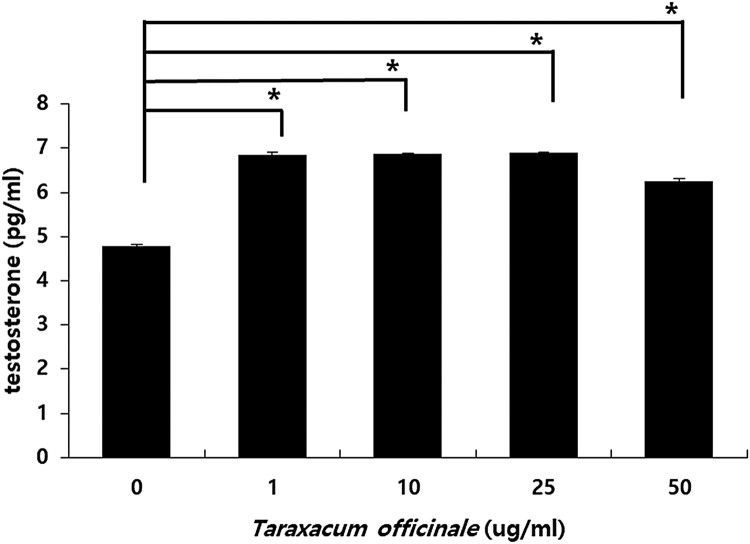
Measurement of levels of testosterone in mouse Leydig cell supernatants. Cells were treated with 0, 1, 10, 25 and 50 µg/ml of *T. officinale* for 48 h. The cell supernatant were collected and used to do the ELISA method. Each group's *p*-value was calculated. (*p < .05, **p < .001). The experiments were performed in three times, respectively.

Mouse Leydig cells were treated with TOE for 48 h. The cell supernatant was collected after centrifugation and removing the cell debris. The cell supernatant was used for analysis using enzyme-linked immunosorbent assay (ELISA). The testosterone levels increased significantly after treatment with TOE at 1, 10, 25, and 50 µg/mL (Figure 5). Thus, the TOE directly increased the testosterone levels, which showed that the TOE is associated with the synthesis of testosterone in vitro.

## Discussion

**Figure 6.**
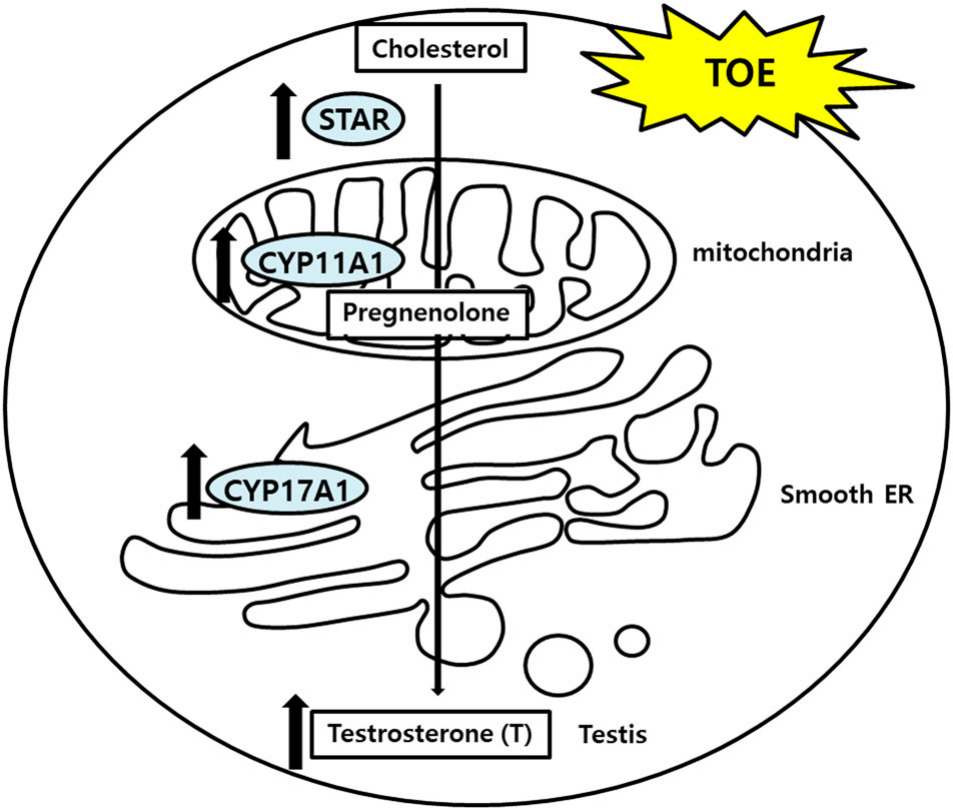
The model of steroidogenesis with the treatment of TOE in mouse Leydig cell.

*T. officinale* has been used in different cultures for its medicinal properties. TOE exerts hepatoprotective effects on sodium dichromate-induced liver injury in rats (Hfaiedh et al. 2016), decreases adipogenesis in 3T3L1 adipocytes (Gonzalez et al. 2014), has immune-enhancing effects in mice (Lee et al. 2012), anti-inflammatory effect on the central nervous system (Kim et al. 2000), and protective effects against lipopolysaccharide-induced acute lung injury in mice (Liu et al. 2010). Plants belonging to the genus Taraxacum are found abundantly in South Korea. We determined the effects of TOE on the levels of testosterone by using TM3 mouse Leydig cells. Our results showed that TOE may be used for the treatment of LOH or for improving spermatogenesis in aging men. In a previous study, we showed that extracts from the dandelion plant increased the survival of TM3 cells and protected TM3 cells against H_2_O_2_ induced cellular stress in vitro. Treatment with dandelion extract (50 µg/mL) increased the cell viability (Noh et al. 2012), and further studies are required to establish the potential of this extract for alleviating symptoms of andropause. However, no study has reported the steroidogenic effects of TOE in Leydig cells in vitro. This study aimed to elucidate the steroidogenic effect of TOE by determining the mRNA and protein levels of steroidogenic genes and the levels of testosterone. Our results showed that the TOE increased testosterone biosynthesis by increasing the expression levels of steroidogenic enzymes such as STAR, CYP11A1, and CYP17A1. We determined the mRNA (Figure 3) and protein (Figure 4) levels of these enzymes and the levels of testosterone using cell supernatants (Figure 5). Several enzymes are involved in steroidogenesis in mouse Leydig cells (Zhong et al. 2013; Beattie et al. 2015; Ohta et al. 2017). Binding of LH to the LH receptors results in the formation of luteinizing hormone-choriogonadotropin receptor (LHCGR) complex. The LHCGR complex stimulates cAMP to use cholesterol in the cytosol as a substrate for steroidogenesis, and cholesterol activates STAR, a cholesterol transporter (Beattie et al. 2015). Our results showed that the mRNA (Figure 3(C)) and protein (Figure 4) levels of STAR increased in a dose-dependent manner after treatment of mouse Leydig cells with TOE for 24 and 48 h, respectively. Thus, TOE induced the expression of STAR in vitro. STAR and cholesterol form cholesterol-STAR complex. The TSPO present in the outer mitochondrial membrane facilitates the transport of cholesterol to the inner mitochondrial membrane of Leydig cells, which regulate the downstream steroidogenic enzymes present in the mitochondria (CYP11A1, HSD3B) and in the smooth endoplasmic reticulum (HSD3B, CYP17A1, and HSD17B). Treatment of mouse Leydig cells with TOE increased the mRNA (Figure 3(A,B)) and protein (Figure 4) expression levels of CYP11A1 and CYP17A1. Thus, TOE increased the expression of CYP11A1 in the mitochondria and CYP17A1 in the smooth ER. Further, we examined the biosynthesis of testosterone as a last product during steroidogenesis (Luo et al. 1996; Luo et al. 2001; Culty et al. 2002; Luo et al. 2005; Beattie et al. 2015). We determined the testosterone levels by performing ELISA using a cell supernatant obtained after treatment with TOE for 48 h. The synthetic testosterone levels with the treatment of 1, 10, 25 and 50 µg/mL of TOE are increased significantly. Especially, the testosterone levels in the group treated with 25 µg/mL of TOE, 6.885 pg/mL, were significantly higher than in the control group, 4.782 pg/mL (Figure 5). Thus, TOE has steroidogenic effect and we proposed the model of steroidogenesis in mouse Leydig cells (Figure 6).

Our results of the effects of TOE in improving spermatogenesis or LOH were consistent with those obtained using other medicinal plants. Some flavones such as chrysin (Jana et al. 2008) and apigenin (Li et al. 2011) increase androgen production. Luteolin (10 μM), one of the flavonoids, stimulates cAMP-dependent StAR, Cyp11a1, and Fdx1 promoters and may increase steroidogenesis in MA-10 Leydig cells (Cormier et al. 2017). Eurycomanone increases spermatogenesis by inhibiting the aromatase-mediated conversion of testosterone to estrogen in Leydig cells (Low et al. 2013). A previous study showed that 20 mg/mL of *Cordyceps sinensis* had an effect on steroidogenesis by increasing the protein levels of cAMP and StAR in MA-10 mouse Leydig cells (Huang et al. 2000).

Pharmacological effects of many plants have been studied. We established the steroidogenic effect of TOE for utility in improving male health. However, the safety and efficacy of plant preparations or extracts are unknown, thus limiting their use. The active agents in plant extracts are not well defined and sufficient information about the toxicity and adverse effects of these plant preparations is not available. In addition, no information is available about the pharmacokinetics and bioavailability of such extracts. Thus, to prove the medicinal plants, the first the safety, precise dosage or usage and efficacy were assured before usage. The second, the selected plants have been processed such as quality control, standardization, and validation with various methods. These procedures are required to separate the crude extract and obtain a fine extract. Thus, further studies are required to establish the safety, quality, and efficacy of medicinal plants and plant preparations used as natural materials.

## Conclusion

We showed that the TOE increased the mRNA and protein levels of steroidogenesis-related enzymes CYP11A1, CYP17A1, and STAR and increased the production of testosterone in mouse Leydig cells. Thus, the TOE may be used as an alternative medicine for the treatment of diseases characterized by insufficient testosterone, such as male infertility, hypogonadism, and LOH. Further studies are required to establish the clinical efficacy of TOE. Our results indicate that TOE might be a promising therapeutic agent obtained from a medicinal plant for increasing steroidogenesis or improving LOH.
